# Automatic Annotation for Human Activity Recognition in Free Living Using a Smartphone

**DOI:** 10.3390/s18072203

**Published:** 2018-07-09

**Authors:** Federico Cruciani, Ian Cleland, Chris Nugent, Paul McCullagh, Kåre Synnes, Josef Hallberg

**Affiliations:** 1 Computer Science Research Institute, Ulster University, Newtownabbey BT370QB, UK; i.cleland@ulster.ac.uk (I.C.); cd.nugent@ulster.ac.uk (C.N.); pj.mccullagh@ulster.ac.uk (P.M.); 2 Department of Computer Science, Electrical and Space Engineering, Luleå University of Technology, 97187 Luleå, Sweden; kare.synnes@ltu.se (K.S.); josef.hallberg@ltu.se (J.H.)

**Keywords:** human activity recognition, supervised machine learning, label noise, automatic annotation, inertial sensors, smartphone

## Abstract

Data annotation is a time-consuming process posing major limitations to the development of Human Activity Recognition (HAR) systems. The availability of a large amount of labeled data is required for supervised Machine Learning (ML) approaches, especially in the case of online and personalized approaches requiring user specific datasets to be labeled. The availability of such datasets has the potential to help address common problems of smartphone-based HAR, such as inter-person variability. In this work, we present (i) an automatic labeling method facilitating the collection of labeled datasets in free-living conditions using the smartphone, and (ii) we investigate the robustness of common supervised classification approaches under instances of noisy data. We evaluated the results with a dataset consisting of 38 days of manually labeled data collected in free living. The comparison between the manually and the automatically labeled ground truth demonstrated that it was possible to obtain labels automatically with an 80–85% average precision rate. Results obtained also show how a supervised approach trained using automatically generated labels achieved an 84% f-score (using Neural Networks and Random Forests); however, results also demonstrated how the presence of label noise could lower the f-score up to 64–74% depending on the classification approach (Nearest Centroid and Multi-Class Support Vector Machine).

## 1. Introduction

Human Activity Recognition (HAR) is a major area of study and, with the growing diffusion of mobile devices, the past few years have witnessed the use of smartphone-based approaches to HAR becoming increasingly popular. Although sensor based HAR in general is an advanced field of investigation, there are still open challenges to be addressed. In addition, smartphone-based solutions, while representing an opportunity, also introduce new challenges. Some of the assumptions generally valid in sensor based HAR may be false when approaching the problem using the smartphone, for instance, the assumption that location and orientation of the sensor are known a priori (as in the case of wearable sensors) [1,2]. Moreover, different users may have different ways of carrying the smartphone with them.

Besides these new set of challenges, all obstacles common to any HAR approach have to be faced. Specifically, solutions are (in most cases) trained offline, and often using data exclusively collected in a controlled environment [2]. An analysis of performance of these solutions has shown how accuracy levels can drop significantly when moving to free-living environments [3,4]. Another problem resides in the lack of personalization. Solutions trained offline on a subset of users’ data have been shown to perform with a lower accuracy rate on unknown users (i.e., where their data has not been used to train the classifier) [1]. Consequently, research interest towards *online* approaches to HAR (i.e., methods able to implement a training mechanism that can be performed locally and in real time) has recently been growing [2]. The biggest obstacle to online methods, however, is the need for labelled data to be used both as data points for training models, and as a ground truth dataset to validate their performance [5,6]. This is a general problem for all supervised learning methods, which not only require the presence of a large dataset, but also require human supervision to annotate the dataset. Despite this limitation, supervised methods represent the most common approach to HAR, since achieving HAR in a completely unsupervised fashion may be a difficult task to be accomplished [7].

Many solutions have been developed to support and facilitate the process of data annotation [5,8,9]. Similar systems can significantly reduce the time required by the data labeling process. Nevertheless, these mechanisms are viable only for offline training methods, where models are trained beforehand on a limited number of subjects and are not viable for online and personalized approaches. In other studies, such as [10], crowd labeling solutions of prompting users in order to generate labels have been proposed. This facilitates the collection of data in a semi-automatic fashion where the user either confirms/corrects a label generated automatically or directly provides a new annotation. Crowd labeling approaches facilitate the development of online and personalized training; however, user interaction is still required to label the data, making the process time-consuming and potentially invasive from the user’s perspective. Moreover, crowd labeling approaches can introduce labeling errors either due to mistakes or by the user misinterpreting the meaning of a label [11].

An alternative approach to reduce the need for labelled data points is to consider semi-supervised methods that apply methods such as label propagation allowing a classifier to be trained using a dataset of which only a small percentage data points are labelled [12]. In either case, human interaction is still required to produce a first level of annotation.

As an alternative to data-driven methods (both supervised and unsupervised), knowledge-driven methods have also been proposed. These approaches aim to build activity models based on a-priori information, thus not requiring the presence of labelled dataset [13]. Despite the main advantage of not requiring annotation, these models in general are not robust to data uncertainty and/or to inter-person variability [13]. To overcome either methods’ limitations, hybrid approaches (combining knowledge and data-driven) have subsequently been proposed [13].

In this work, we target the data labeling problem by proposing a knowledge-driven automatic labeling method that enables an online supervised training approach. The training phase is encompassed using *weak* labels generated automatically by a heuristic function combining step count and GPS information. The final classification is performed online and locally using only data from the accelerometer. In ref. [14], we explored the viability of a supervised approach relying solely on automatic labeling of the training dataset in the form of weak labels to train an accelerometer-based classifier. Although encouraging, preliminary results have shown how label noise (produced by the heuristic) affects the final recognition accuracy. In particular, a larger number of samples is required to reach acceptable levels of accuracy, and, even when collecting a larger dataset results obtained, are less accurate when compared to the same classification approach using a manually labeled dataset. We present here a new heuristic function that is able to generate (along with weak labels) a probability estimation of the label quality. This information can be beneficial when balancing the dataset across the list of activities of interest, allowing the removal of a labeled set of samples believed to be less reliable, while keeping data points more likely to contain higher quality annotations. Using the improved heuristic version, an overall accuracy of 84% has been observed against the initial 74% accuracy obtained in ref. [14].

This work presents a novel method for the automatic labeling of a dataset that reduces the burden of generating a training dataset. In addition, results present a comparison of accuracy performances, examining robustness of conventional Machine Learning (ML) approaches to label noise, which may be relevant not only for HAR using automatic labeling.

The remainder of this article is structured as follows: Section 2 summarizes relevant literature, the state of the art and limitations of current approaches. In Section 3, we present a heuristic function used to automatically label acquired data points. The methodology is explained in Section 4. Section 5 describes the dataset used to evaluate the experiment. Results are presented in Section 6. Finally, Section 7 and Section 8 provide discussion of results obtained, and possible directions for future work, respectively.

## 2. Background

This section provides an overview of sensor based HAR and related challenges, providing particular emphasis on smartphone-based and more specifically accelerometer-based approaches. Moreover, while presenting this summary of more common approaches, we wish to highlight the advantages and disadvantages of each approach with respect to the dataset annotation problem, and to online and personalized training. This section summarizes the most common sensor modalities, feature extraction techniques and classification approaches. In addition, we will refer to the most common classes of target activities, known limitations and open challenges for HAR.

### 2.1. Smartphone-Based Activity Recognition

In HAR, the most common sets of target activities include ’sitting’, ’standing’, ’walking’, ’running’, ’cycling’ or ’using some means of transportation’ [1,2]. In some cases, subclasses are also considered such as ’walking up stairs’ and ’walking down stairs’. Some solutions can also consider walking with the phone in pocket, rather than keeping it in the hand as two different classes [2]. In other cases, classes can be representative of a wider spectrum of activities, as in the case of considering a single class ’standing/walking’, or distinguishing macro classes such as ’active’ and ’inactive’ (for instance to monitor sedentary behaviors) [4,15]. Some HAR solutions consider an extended set of target activities that also include some Activities of Daily Living (ADLs) (e.g., cooking, eating or cleaning) [2,12]. This is, however, more common in solutions involving the use of environmental sensors (as for example within a smart home scenario), and only a small percentage of smartphone-based systems have been targeting ADLs [2,4].

The classification task can be performed *offline* (when data processing is performed asynchronously and at a later stage with respect to data collection) or *online* (recognition performed in real-time) [6]. Similarly, the training phase of models can be performed offline or online [2,4]. The combination of offline training and online classification represent the most common approach [2,4]. Offline training is often performed in controlled environments, although moving the solution to a free-living setting has been shown to affect the system causing a significant decrease in the accuracy performance [1,2,3]. Moreover, offline trained solutions have been observed to perform with lower accuracy rates for unknown subjects (i.e., not used for training purpose), and generally solutions present a significant inter-person variability affecting the recognition rate [1,2,6]. On the other hand, *online* training opens the way to personalized solutions. In this case, a pre-trained classifier can be updated when new data (subject specific) become available, in order to reduce the effect of inter-person variability [2]. Personalized training, however, exacerbates the need for labeled datasets for training purposes making the approach not viable using conventional supervised methods.

A generic Activity Recognition (AR) process can be divided into a finite set of stages sometimes referred to as Activity Recognition Chain (ARC) [6]. A typical ARC will contain the following stages: sensing (data collection), pre-processing, feature extraction and classification.

In the following subsections, the most common methods for each of these steps are described. Finally, open challenges for HAR are evaluated.

### 2.2. Sensing Modalities

The range of sensors that has been used in sensor-based HAR is rather wide, including motion, contact and inertial sensors, RFID based systems and audio-based solutions. Similarly, many types of sensors have been considered also in the case of smartphone-based HAR. In this case, however, the choice is limited by the range of sensors commonly on board. Smartphone-based solutions rely, in most cases, on inertial sensors. Inertial sensors (accelerometer and gyroscope) are, by far, among the most common used sensors for a number of reasons:

- they are present in almost all modern smartphone models independently from the model price range;
- they have proved to provide good recognition accuracy rates;
- the data provided do not pose privacy issues (e.g., as in the case of microphones);
- and they are less power demanding compared to other sensors such as GPS.

Accelerometers have been more widely adopted, sometimes in combination with gyroscopes and/or magnetometers [1,2]. Accelerometer extracted features have been shown to be more informative, and the combination with data from gyroscopes and magnetometers has been demonstrated to improve the accuracy by 5% [1]. In addition, the combination of inertial sensors with other sources of information, such as GPS, barometers, and (more rarely) microphones, has been shown to improve accuracy [1,2]. For instance, the combination of accelerometer data with barometer data can provide better accuracy in distinguishing between walking down and up the stairs, compared to an accelerometer-only based solution [1,16]. Even if the combination of multiple sensors can be considered as beneficial in terms of accuracy performance, accelerometer-only based approaches can be considered as optimal in terms of the tradeoff between accuracy rates and resource consumption (required computational complexity and power consumption) [1,2], a fact of particular relevance for smartphone embedded solutions.

### 2.3. Preprocessing

Inertial sensor signals are often preprocessed before feature extraction [1]. In most cases, preprocessing includes a filtering and a segmentation step. In the case of HAR, for instance, a low-pass filter can be used to exclude high-frequency signal components. In fact, for more common target activities, 98% and 99% percent of the information are contained in the 1–10 Hz, and in the 1-15 Hz frequency bands, respectively [1]. Consequently, most common sampling rates for inertial sensors are in the range of 20–30 Hz to avoid undersampling while avoiding unnecessary battery consumption that higher sample rates would require [1]. Segmentation is generally performed using a sliding window approach (either with overlapping or non-overlapping techniques) [1,2]. Window sizes, in most cases, vary in the range of 1–10 s intervals. The optimal window size for feature extraction depends on the set of target activities, however, for common target activities of HAR, a window size of 1 s has been often identified as optimal [1,17].

### 2.4. Feature Extraction

Extracted features can be divided into two main categories: time domain and frequency domain features. Common time domain features include statistical moments of the signal such as mean, variance, skewness and kurtosis [1,2,6]. Other common time domain features are minimum and maximum values in the interval, and distance between peaks [1,6]. Frequency domain features require Power Spectral Density (PSD) calculation (using Discrete Fourier Transform (DFT)), and are therefore more computationally demanding with respect to time domain features [1]. Common frequency domain features are PSD energy and entropy or number and locations of peaks in the PSD [1,2].

In the case of inertial sensors, features can be extracted out of the three channels that three-axis accelerometers and gyroscope provide. Information extracted from the three axes can lead to better accuracy results, especially when location and orientation can be assumed to be known a priori. However, this is not the case for smartphone-based HAR where this particular assumption is not valid, since users may have different ways to carry the smartphone. To deal with this problem, three main alternatives have been explored [1]:

1. to adopt a hierarchical strategy in which a classification has the goal of detecting location and orientation of the sensor, and then an appropriately trained classifier can be used for recognition (this solution, however, exacerbates the complexity of generating the training dataset for all possible combinations) [18]
2. a much simpler approach is to use the 3D magnitude of the signal [19]
3. finally, sign-invariant features (as the absolute value of features on the three axes) can be extracted providing robustness to some orientations and considering only the axis from the top to the bottom of the screen and the one pointing out of the screen [15].

In ref. [6], a comparison of results obtained using different sets of features showed how time-domain features (particularly mean and variance) were the most informative. Nonetheless, in many cases, a combination of time and frequency domain features have been used [1,2,20].

In some cases, the classification process does not involve feature extraction. For example, Deep Learning (DL) approaches can make direct use of raw data for classification [1].

### 2.5. Classification Approaches

Several classification approaches have been used for HAR using supervised methods. In smartphone-based HAR, Decision Trees (DTs), Support Vector Machines (SVMs) and k-Nearest Neighbors (kNN) are among the most common [2]. DT approaches have the advantage of being among the computationally less expensive models, and for this reason their use in smartphone-based applications is relatively common; however, they may not be the best choice for online methods (since the entire model needs to be recalculated when new samples are acquired) [1]. kNN have the advantage of being among the most convenient instance-based methods for online learning, since the adaptation of the model to new data points is simply resolved by storing new calculated features vectors [1]. SVM methods have shown good accuracy results and they share with DT the advantage of not being too computationally expensive, at least at the prediction stage [1]. Probabilistic models such as Hidden Markov Models (HMMs), Conditional Random Fields (CRFs) and Skip Chain CRFs (SCCRFs) have also been used [21,22,23]. Finally, some studies have investigated the use Neural Networks (NNs) for HAR purposes. Although NN models may be computationally demanding (computational complexity can rapidly grow with the number of features in use), they are still viable for online learning approaches, where NN weights can be updated when new data points are available.

As previously mentioned, more recent works have explored DL approaches [1], specifically the use of Convolutional Neural Networks (CNNs) [24,25] and Deep Belief Networks (DBNs) [26]. Although the use of DLs appears to be more accurate, their use may not represent an optimal solution for smartphone embedded solutions due to the required computational complexity. Some studies (such as as [26]), however, have been using DL on the smartphone, at least at prediction stage, for real-time classification.

### 2.6. Classification Accuracy

Overall evaluation of performance is generally presented using precision, recall and f-score [1,4]. Confusion matrices are more often used to show detailed inter-class variability and distribution of false positives across the target classes [1,4].

Past studies in smartphone-based HAR exhibit quite a diverse range of accuracy values between 80% and 97% [4]. These variations can be due both to the dataset used, the classification method, and the specific set of target activities being considered. These variations make the comparison of performances a complex task [1,4]. For instance, unreliable accuracy levels can be measured when the sample size used for testing is too small. On the other hand, increasing the number of samples used for testing can affect performances reducing the size of training data [1]. In a controlled environment, accuracy can reach 97%, although accuracy has been observed to be in the 63–86% for unknown subjects [4]. Similarly, performance variations have been observed according the position/s of the smartphone taken into account. This variation can be avoided by restricting the examined position to the most common case (for example, trouser pocket) [4].

The set of target activities can also impact performance. For instance, in some cases, certain classes have been reported as conflicting with others, thus resulting in lower accuracy levels [1,4]. An example is the biking (or cycling) activity that has been reported in some cases as conflicting with the walking class [4]. Overall accuracy has been observed to vary between 92% excluding cycling and 73% including the cycling activity [4].

### 2.7. Challenges in Smartphone-Based HAR

Although HAR has been an active research field for many years, there are still many challenges to address. One of the main challenges is the need for labelled datasets for ground truth annotation [6]. This challenge is particularly relevant for both online and personalized approaches. Online methods require a way to obtain data labels directly in free-living conditions, while personalized methods further exacerbate the need for data requiring user specific datasets. Another major issue to be faced is class imbalance [6], particularly when collecting data in free-living situations where samples corresponding to some of the activities may be less likely to occur. The process of training using highly imbalanced datasets may alter the final recognition rate significantly, while approaches to balance the training dataset, as in the case of undersampling [27] (i.e., random elimination of data points in the majority class) cause a significant decrease in data available for training.

Data labelling for ground truth generation is a time-consuming task posing a major obstacle to all supervised methods. To deal with the problem, many approaches have been proposed. An example is the development of annotation tools that facilitate manual annotation attempting (at least partially) to automate the process [8,28]. These tools can drastically reduce the effort required to annotate large amounts of data; however, they can only be used in offline conditions. Moreover, annotation tools often rely on video footage for annotation [8], making their use less frequent in free-living settings due to inherent privacy issues.

Crowd labelling approaches have been proposed, particularly in the case of the smartphone, where final users can directly annotate data fragments [10]. Nevertheless, human interaction is still required at some stage to produce the labelled dataset. This results in the process being potentially time-consuming from the user perspective and poses the problem of label accuracy [12]. Semi-supervised methods have been proposed to deal with this problem, however, mostly in other domains [4]. Semi-supervised methods require only a subset of samples to be labelled. With this, they can still make use of unlabeled data points in the training process [12,29]. Common approaches are label propagation or Multi-Instance Learning (MIL), where labels are assigned to sets of instances rather than to single data points [29,30]. The semi-supervised approach has, however, rarely been applied to HAR [4], as in [12]. Moreover, although these methods can reduce the required time effort to produce ground truth data, partial manual annotation is still required. Table 1 summarizes some recent HAR approaches using inertial sensors. The comparison highlights how the problems of data annotation and label noise have been rarely addressed.

In most cases, studies assume availability of a fully labeled dataset. Only refs. [12,36] have tackled the annotation problem by means of semi-supervised approaches requiring partial annotation. In ref. [10], user solicitation is proposed to generate labels online; however, in ref. [12], the use of prompting has been combined with label propagation to minimize the required amount of annotation. In ref. [36], an alternative approach has been proposed to reduce the required amount of labeled data, aiming to exploit activity models trained on other users. Similarly, only [12,34] have examined accuracy performance considering label noise.

## 3. Implementation

The goal of our research is to investigate novel methods to manage the data annotation problem, thus reducing the burden of ground truth generation and opening the way to online and personalized training methods. More specifically, we present an online annotation method aiming to solve the problem of ground truth generation by means of automatic labeling. Activity monitoring is performed using the smartphone and aiming to generate labels outdoor and indoor combining accelerometer and GPS information.

Our study explores the use of *weak labels*, i.e., noisy labels generated automatically using a heuristic function. The heuristic function combines GPS and step count information in a knowledge-driven model, in order to generate the weak labels that are then used to build the training dataset, thus enabling an online learning approach in a free-living setting.

### 3.1. Online Training Architecture

**Figure 1:**
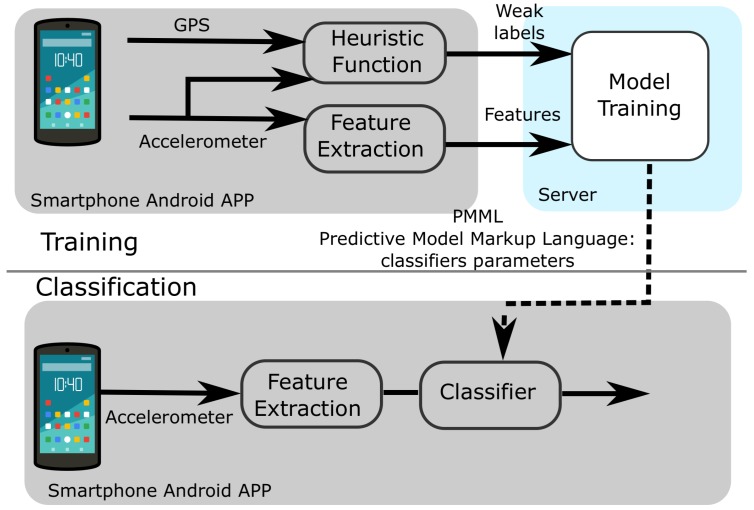
The smartphone app collects raw data samples from the on-board accelerometer and the GPS. The GPS is combined with the step count to generate weak labels using the heuristic function. Extracted features are sent to the server along with the labels in order to train the model. Parameters of the classifier are sent back to the app in Predictive Model Markup Language (PMML) format. Finally, the classification can be performed locally on the smartphone instantiating the appropriate classifier based on the parameters.

As in ref. [14], we propose an online training solution that relies on part of the process to be performed on a centralized server application. Specifically, while data collection and final classification are performed locally and in real time on the smartphone, the training phase is performed remotely (server-side). As depicted in Figure 1, the smartphone collects and send raw data to the server along with heuristic generated weak labels. On the server side, the training phase starts whenever a sufficient number of data points have been collected. Finally, parameters of the trained model (in Predictive Model Markup Language (PMML) format) can be sent back to the smartphone to instantiate the trained classifier to perform HAR locally.

This solution provides two main advantages:

1. it reduces the burden of performing the training on the smartphone, performing this computationally expensive task remotely,
2. it allows for comparing performance (offline) with different classification methods and feature sets at a later experimental stage.

A more efficient scheme following the same architecture can be obtained, however, by sending only extracted features, instead of raw data signals as we did in the current work, to leave the possibility of experimenting using different features sets.

### 3.2. The Heuristic Function for Automatic Labeling

The heuristic function combines step count and GPS information in order to label data samples. Step detectors are often directly available in modern smartphones. In cases where a step detector is not present on board, an estimation of the step count can be obtained using a peak detection approach based on the magnitude of the 3 axes of the accelerometer signal [14]. The GPS is also among the most common on-board sensors in smartphones, and major mobile operating systems allow easy retrieval of the GPS coordinates along with an estimation of the location accuracy.

In ref. [14], we presented an initial version of the heuristic function combining GPS data and step count information in order to automatically label data fragments. This previous experiment highlighted how a supervised approach (trained using weak labels) affects the overall accuracy in comparison with a fully supervised approach trained using a manually labelled dataset. The difference is related to the fact that the heuristic generates an annotation at a coarse level. This is due to different acquisition rates (GPS info update less frequently) that result in higher uncertainty of weak labels compared to the manual labelled ground truth. Preliminary results in ref. [14] have also shown different learning curves between the manually labeled and the automatically generated labels. The fully supervised approach (using manually labeled dataset) converges more rapidly to an acceptable accuracy rate. This is reflected in a significative gap in the overall accuracy, particularly when the training dataset is small; however, the gap is partially reduced using a larger number of samples for training.

In this work, we present an improved version of the heuristic that allows an estimate of the reliability of the generated weak labels that can be used to optimize data points selected for training. This section describes the heuristic function, and how it estimates the *quality* of labels. We then describe selected features, and the classification approach. Moreover, in this paper, we present results calculated over a larger dataset and targeting a different set of activities. In this case, we define the target activities as ’sitting’, ’walking’, ’running’, ’cycling’ and ’transportation’ (while the study in ref. [14] was excluding ’running’ and ’cycling’).

As in ref. [14], a manually labeled ground truth dataset has been collected for validation. The comparison between the ground truth (manually labeled) and the weak ground truth (labeled automatically) provides information on the quality of labeling of the heuristic, which constitutes essential information in order to evaluate the final accuracy rate.

Step count is used as metric for a first heuristic rule, which can detect a number of activities based on the steps/minute (spm) rate as in [14]. A walking pattern corresponds typically to a pace of 90–110 spm [39,40]. Similarly, a running stride normally resides in the 160–180 spm range [41]. Having the step count information, the spm rate is sampled on a 1 min fragment. This window allows the first level heuristic to deduct, for instance, that a consistent 102 spm rate in a 1 min window will most likely correspond to a ’walking’ pattern. The second step is a merging phase. Consecutive time windows are merged when the difference between the two is less then 10 spm. This allows detection of prolonged walking sessions (e.g., longer than 10 min) and prolonged time with spm 0 or close to zero, which the heuristic will label as sedentary. The merging phase is particularly useful when combining the step count information with the GPS, since the GPS is sampled at a lower sampling rate (polled at variable intervals between 1–3 min) to minimize the impact on battery consumption. The second step of the heuristic considers GPS locations within the merged interval. Consecutive timestamped locations are used to estimate user speed. If multiple speed estimations are present in the merged interval (obtained from the step count), the heuristic will calculate the weighted average speed on the merged interval. As in the case of the step count, estimated speed can be used to recognize the most probable locomotion pattern in outdoor activities (≃1.4 m/s walking, 3–4 m/s running, cycling 4–8 m/s and using some mean of transportation ≥20 m/s) [41,42]. The combination of the two information sources can reduce the presence of wrongly assigned labels. For instance, a measured speed comparable with riding some means of transportation allows the algorithm to ignore a false positive in a step count. Similarly, a consistent running spm rate can identify a running on a treadmill activity where the GPS would not produce any valuable information.

In ref. [14], we directly assigned labels merging the step count and GPS information. In this enhanced version of the heuristic, a probability estimation is introduced aiming to assess the *quality* of produced weak labels using a probability model.

#### Assessing Quality of Labels

**Figure 2:**
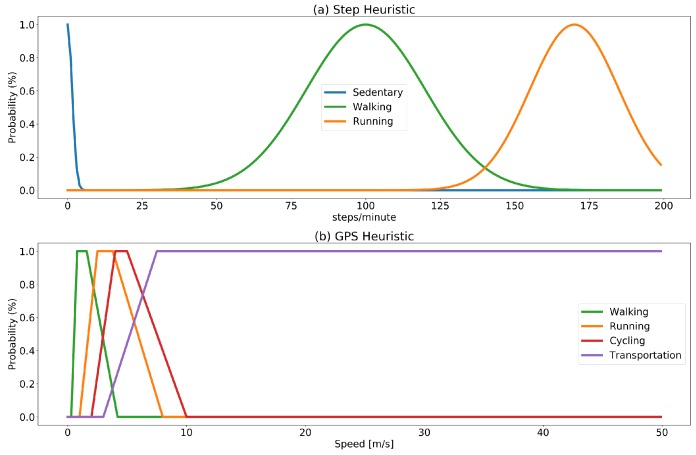
Fuzzification of probabilities values for the heuristic function. Step count heuristic (**a**) is modeled using Gaussian membership functions with average and standard deviation based on common steps/minute rates for walking and running. Similarly, (**b**) shows the trapezoidal membership functions used to estimate probability based on measured speed in m/s, for walking, running, cycling and using some means of transportation.

The probability assigned to a weak label is calculated by merging probability functions corresponding to the target activities, modeled as *membership functions* of a fuzzy set. Fuzzification of values provides a convenient way to map values (steps/minute rate and estimated speed) into a probability model. The set of target activities is modeled into two fuzzy sets: one for the step rate and one for the GPS as depicted in Figure 2. The set of membership functions for the step count is modeled using Gaussian distributions (with mean and standard deviation centered in the normal walking and running rate). The choice of modeling step count probability functions using a Gaussian distribution is based on the fact that walking and running rates are assumed to be normally distributed in the population. The spm rates for walking and running intervals are taken from [39,41]. The set of membership functions for the GPS estimated speed is built using trapezoidal functions shaped considering estimated speed. The choice of trapezoidal functions is based on the fact that the assumption of estimated speed being normally distributed cannot be made in this case, since speed is affected by multiple factors (e.g., vehicles in use and traffic conditions).

The two fuzzy sets are used to generate two labels predicting the most probable ongoing activity independently using GPS and step information with a probability estimation for the two predicted labels $P_{GPS}\left(label\right)$ and $P_{step}\left(label\right)$. The membership functions used to estimate probability, are designed to produce low probability results (below 50%) for a range of values that could be ambiguous. For instance, a rate of 50 spm over a five-minute interval could either be produced by continuous walking at a very low pace, or it could be the case that the interval includes some walking and some sitting. Similarly, a rate of 130 spm could correspond to an interval including some walking and some running.

The sets of activities that the two information sources are able to detect do not coincide; i.e., the step count will be able to detect walking, running or to estimate sedentary intervals (as prolonged interval without an increase in the step count); however, it will not be able to provide valuable information to discriminate activities such as cycling or using some means of transportation where the GPS will be more informative. The estimation of final probability, therefore, distinguishes the cases of labels that can be detected by both information sources (sitting, walking, running) and activities that can be detected only by using the GPS. In the first case, the final probability will be estimated by combining the probability obtained from the two set fuzzy sets performing the weighted average of the two probability values:

(1) $$P\left(Walk\right)=\frac{P_{step}\left(walk\right)+w_{GPS}P_{GPS}\left(walk\right)}{1+w_{GPS}}.$$

The weighted average allows for using another piece information which is the accuracy of the GPS location. The weight of the GPS prediction ($w_{GPS}$) will be proportional to the accuracy rate of the two locations used to calculate the estimated speed. In this way, the GPS will be more informative in outdoor situations where the accuracy can be expected to be higher. For other cases, as discriminating between transportation and cycling, the produced label will be assigned based on the probability obtained from the GPS. Discriminating cycling and driving activities only using GPS may not be a reliable method in cases such as driving in traffic where the two activities can have comparable speeds. To reduce the presence of mislabeling of this type, after the annotation is generated, the final step of the heuristic is to consider the label generated at the previous interval, converting a consecutive sequence of the type ’Cycling’-’Transportation’, or ’Transportation-Cycling’ as ’Transportation’.

**Figure 3:**
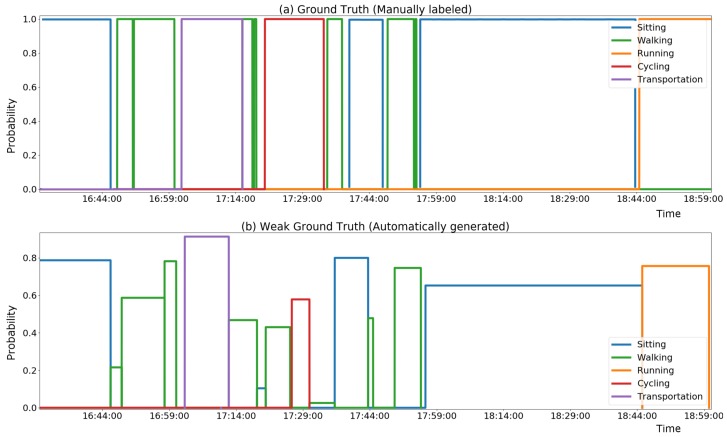
Comparison of ground truth (**a**) with heuristic generated annotation (**b**).

Figure 3 presents an example of the annotation produced by the heuristic, in comparison with the manual annotation.

The heuristic can be expected to provide reasonably good precision (ratio of correctly assigned weak labels) under certain conditions, such as good GPS signal and occurrence of prolonged consistent activities. On the other hand, the approach is expected to have low recall performance (missing labels). This is because, in other cases, the heuristic will not be able to predict labels with high enough reliability (e.g., presence of high variability as alternating different activities, and/or poor GPS signal). Therefore, our approach is to use the heuristic information for data mining of a weakly labeled dataset, rather than use the heuristic directly to classify the ongoing activity. The automatically labeled dataset can then be used to train a classifier based using only inertial sensors’ features, which will be able to classify the on-going activity continuously and also in cases where the heuristic would not be informative.

### 3.3. Segmentation and Feature Extraction

Accelerometer data is sampled at 30 Hz. Segmentation was performed using a sliding window approach with a 50% overlap and 1 second as window size. Our approach mostly relies on time domain features; however, it includes some frequency domain features. The complete set of features as in Table 2 includes statistical moments (mean, variance, skewness and kurtosis) extracted from the 3D magnitude (square root sum of the three axes) of the accelerometer signal, and mean, variance and range (max-min difference) at a single axis level. Frequency domain features are computed using the Fast Fourier Transform (FFT) to compute the PSD of the signal; then, the features extracted consist of the number of peaks in the PSD and location of the dominant peak.

## 4. Methods

In this experiment, we compared common classification approaches, prioritizing models that are less computationally demanding at the prediction stage. This comparison has the goal of identifying the best candidate that can be used to perform classification in real time and locally on a smartphone, when dealing with a training set that may contain noise.

### 4.1. Experimental Protocol

**Figure 4:**
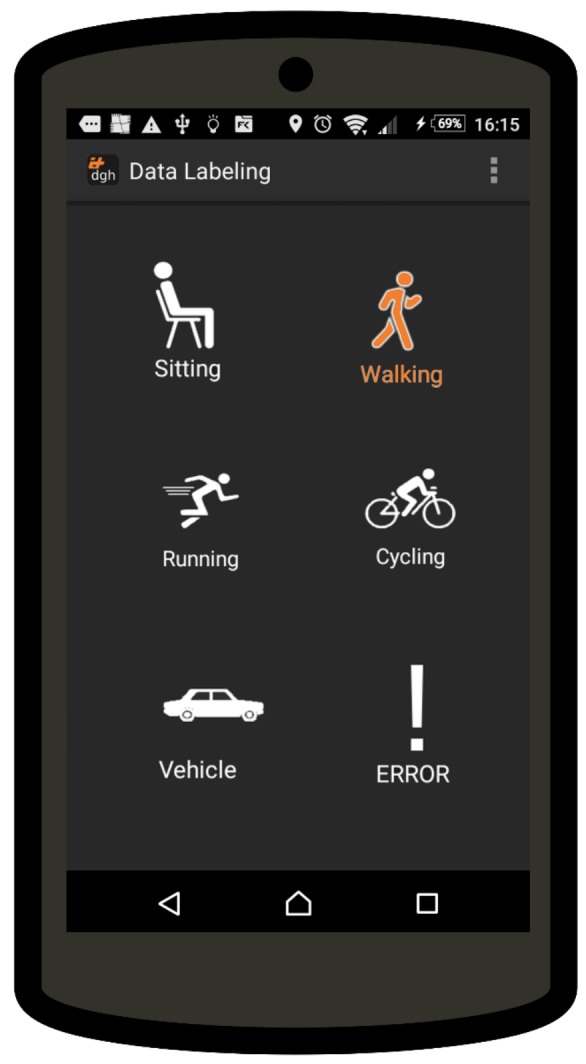
Screenshot of the labeling app showing the buttons to label activities with the ’Walking’ activity selected as currently on-going. The error button allows the user to signal whenever an error occurs in the labeling, allowing for ignoring incorrect annotations for the final evaluation.

As in our previous experiment, the dataset has been manually labeled using a mobile app. The app has been installed on the user’s personal smartphone. The user has been told to carry the smartphone as he would normally do. The app starts collecting data from GPS and the accelerometer in the background after first launch, with no further interaction needed for data collection purposes. The user was asked to use the app to annotate activities while performing his normal routine, allowing to have a manually labeled groundtruth to assess both the accuracy of the automatic labeling, and the final detection accuracy of a trained classifier. The user interface of the app has been designed to be intuitive, and no further training was required. The annotation process is done in real time while activities are being performed. The annotation is performed in the following way. The user selects the start of an activity clicking a label, and this activates the selected label on the interface. Labels are considered as mutually exclusive; therefore, the start of an activity would result in closing the one previously activated (if present). As depicted in Figure 4, the interface provides icon buttons for target activities and an error button to notify the presence of an error in the labeling (e.g., user forgetting to activate the new activity in the transition). The error label is a special label in the dataset. This method allows the algorithm to ignore the last label preceding the error notification when generating the final ground truth. Moreover, to reduce the local uncertainty of the labeling process, a window of 4 s (2 before and 2 after the manually labeled transition) has been ignored between two consecutive activities.

### 4.2. Data Analysis and Validation

Given that data collection is performed in free-living, the data collected is expected to produce a highly imbalanced dataset, where the sitting class can be expected to be the majority. We address the problem of balancing the training dataset using an undersampling approach [27]; however, instead of performing random elimination, the estimated quality of labels is used to eliminate those samples believed to be less reliable. The balancing algorithm divides the data points following the partition set that the heuristic generates. All samples in each group are then sorted by the estimated probability that the heuristic provides, leaving at the top samples believed to be more representative. Finally, only the first *n*-samples are considered where *n* is defined considering the class with the minimum number of samples. The canonical undersampling approach would define $n=n_{min}$, where $n_{min}=min(|C_{1}\left|,\right|C_{2}\left|,\right|C_{3}\left|,\right|C_{4}\left|,\right|C_{5}\left|\right)$, i.e., *n* is equal to the minimum cardinality of the five classes corresponding to the target activities (’sitting, ’walking’,’ running’,’cycling’ and ’transportation’). Instead, we allowed up to 30% more samples with respect to the minimum class. Therefore, for each set, the number of samples used for training would be:

(2) $$n_{i}=min(|C_{i}\left|,n_{min}*1.3\right).$$

Once the number of samples has been balanced across classes, we can generate the final dataset, where each sample will have the form depicted in Table 3. The first column is defined as the label manually collected, the second column contains the weak label (generated by the heuristic), followed by the features defining that sample. Although the data has been collected continuously, we restricted the dataset here considering only data portions for which manual labeling was available.

The validation routine repeats the following steps in a 10-fold cross validation:

1. balanced samples sets are randomised and divided into a train and test set (10%),
2. the training set is obtained by merging and shuffling the sets and using the weak labels for training,
3. scoring of the classifier is performed on the test set using the labels manually collected.

## 5. Dataset

The dataset consists of 38 days of continuous recording of smartphone sensors in free-living, including raw data samples of accelerometer and GPS. The heuristic function was able to generate 200 h of raw data with weak labels. The manually labeled data portions consist of a total of approximately 100 h. Annotation has been performed in free-living over the 38 day period, whilst performing normal ADLs such as preparing/having breakfast, commuting to work and recording physical activity during working hours. Table 4 summarizes the total labeled time and number of samples (with a 1 s window size) for all target activities.

The accelerometer has been sampled at 30 Hz, while GPS sampling was scheduled on a 1–3 min periodic sampling to reduce the impact on battery consumption. Data have been collected extending the dataset in ref. [14] on one subject researcher. The position of the smartphone has been constrained to be the most common (trouser pocket). The training dataset obtained after balancing samples across the target activities consisted of about 2 h and 50 min (about 30 min per class). Considering the size of such a dataset, in this specific case, the last 10 days of recording are sufficient to generate a training set of equivalent size. The number of required days of observation, however, depends on how often the specific user performs the target activities or, in other words, on how activities are evenly distributed.

## 6. Results

This section describes results obtained in our experiment, providing first an analysis of the accuracy measured on labels generated by the heuristic function; then, we provide results obtained by comparing different classification approaches to evaluate how the final accuracy is affected by the initial uncertainty of labels.

### 6.1. Heuristic Accuracy

**Figure 5:**
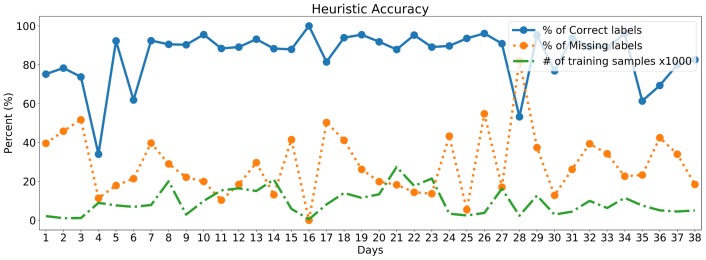
Accuracy of heuristic function over the 38 days composing the dataset. Label precision has been calculated as the number of correct labels divided by the total number of weak labels. Percentage of missing labels has been calculated as the number of missing labels divided by the number of total labels manually annotated.

Analyzing the agreement between the manually labeled ground truth and the one generated using the heuristic, it is possible to measure the precision of the heuristic, as a measure of quality of the produced labeling. Figure 5 presents the performance of the heuristic evaluated over the dataset, highlighting the percentage of correct labels, the percentage of missing labels (in which the heuristic could not provide a label) and finally the number of candidate samples for data training. The accuracy of the heuristic (percentage of correctly assigned labels) is quite variable. This is because we can expect the heuristic to work better in situations with lower variability (e.g., presence of prolonged activities such as a long walk), rather than high variability situations alternating small fragments of different activities.

This accuracy fluctuation, however, is (at least partially) mitigated using the information on probability, resulting in a smaller number of samples actually considered as good training data points, when the overall accuracy decreases. The average precision rate of the heuristic despite fluctuations has been observed to be around 85%. On the other hand, the percentage of missing samples provides an idea of the heuristic recall, confirming that, in some cases, the heuristic is not able to generate a label in more than 50% of the cases.

### 6.2. Performance Evaluation

The evaluation of accuracy performance has been performed by comparing different training algorithms including: DTs, Random Forests, kNN, Nearest Centroid, multi-class SVM and NNs. NNs have been observed using different topologies (with one and two hidden layers) and using different *alpha* values (that can help to avoid or reduce overfitting in some cases). Table 5 presents performance measured in terms of precision, recall and f-score, values averaged on the set of target activities.

Table 6 depicts the confusion matrix for the classifier with the highest f-score (an NN model using two hidden layers with network topology 18 × 36 × 12 × 6). The confusion matrix provides a more detailed visualization of how error rates are distributed over the target activities, and highlights which activities are more often confused.

## 7. Discussion

The analysis of the quality of the automatic labeling confirms a high variability, directly observable in the precision score of the function heuristic itself. Days with high variability (i.e., with frequent alternations between different activities in a short time) exhibit a lower percentage of correctly assigned labels. The probability estimation, however, allows the algorithm to detect the phenomenon under certain conditions, resulting in a smaller number of samples considered as good candidates for the training set. The observed average precision is between 80–90% meaning that label noise can be expected to affect 10–20% of the labels (considering the entire dataset), while label noise measured on the balanced dataset (used for training) appeared to be reduced by approximately 10%. The results highlight how some methods appear to be more robust to this label noise. It can be expected that instance-based methods can be particularly sensitive to mislabeled samples. In particular, for Nearest Centroid, mislabeled samples appear to decrease the accuracy, an effect that is partially mitigated with kNN. Similarly, DT presents high variability in f-score examining the 10-fold cross validation iterations, while Random Forests appear to be more robust. NNs (together with Random Forests) scored the highest f-score values, obtained using higher values of alpha, which reduces likelihood of overfitting, encouraging smaller weights in the network. Overall, the two methods showing the best results are NNs and Random Forests. These two methods appear to be more robust to label noise and show similar values in both precision and recall. These values are also in line with the average precision of the heuristic as could be expected. Although the two methods have similar performance, NNs may be identified as a better option because of their ability to support online training though partial training (i.e., updating the weights when new samples are available). With both NNs and Random Forests, the trained model appears to preserve the initial heuristic precision, while at the same time augmenting the overall recall, since the model (in contrast with the heuristic) will be able to generate predictions continuously. In other cases (Multi-class SVM and Nearest Centroid), label noise appears to impact finally accuracy, resulting in a decrease in precision between 3% and 15%. Moreover, label noise is not evenly distributed across classes. Samples in the ’transportation’ class are sometimes labeled as ’cycling’ because of the speed measured on the GPS (as in the case of driving in traffic). Examining the confusion matrix, it can be observed how this puts into conflict the transportation and the cycling class. This is a direct drawback of the choice of not sampling the GPS continuously to reduce the battery drain. Nevertheless, a more accurate labeling can be achieved using continuous GPS monitoring. Similarly, in some cases, the ’walking’ class is in conflict with the ’cycling’ class. In this case, the difference can be only partially attributed to some incorrect labeling; however, it can also be attributed to a features set generating similar samples for the two classes in some cases.

Overall, this enhanced version of the heuristic appears to improve significantly the final classification accuracy. In particular, the substitution of random elimination (as performed in a previous experiment [14]) with the elimination of samples, considered to be less reliable, improves the quality of labeling of the training set by reducing label noise.

Results obtained appear to be in a similar range of accuracy of other related works, as can be observed comparing accuracy with similar studies reported in Table 1. The comparison in terms of performance, however, is a hard task, due the fact that other studies may consider a different set of target activities and/or do not consider label noise. On the other hand, our approach requiring the presence of GPS data together with inertial data did not allow for comparing results on publicly available datasets because, in most cases, GPS data is missing and/or data have been collected in a controlled environment. Another limitation of this study is that the dataset (being single user) does not allow to assess inter-person variability or to assess accuracy of personalized methods against the approach using one classifier for all users. Instead, the focus of this study has been limited to assessing viability of an online training approach reducing the burden of generating an annotated dataset by means of automatic labeling. This represents a necessary step on the way towards personalized approaches and will also facilitate the collection of a multiple-user dataset in the future.

## 8. Conclusions

With respect to the previous study [14], the estimation of a label’s quality appears to increase the overall accuracy significantly. In particular, replacing random elimination of samples with a heuristic based approach appears to reduce the noise introduced by the automatic labeling. The results obtained demonstrate how an online training approach can be achieved avoiding the burden of manual labeling of data, potentially facilitating the development of personalized systems. In ambiguous cases, it could be useful to prompt the user to verify the correctness of labels. This functionality could be restricted only to some specific cases (e.g., ambiguity between ‘cycling’ and ‘transportation’) to avoid unnecessary user solicitations. In a similar way, some optimization mechanism can be developed to adapt the probability estimation of labels to the specific user. This adaptation can help to distinguish users exhibiting different walking patterns. A simple way to realize the adaptation could be performed by centering the Gaussian membership function of the fuzzy set, around the normal walking rate of the specific user.

In this paper, we proposed an automatic labeling approach to deal with the data annotation problem. An estimation of the quality of the labeling produced has also been proposed, and the use of such information has proven to be useful when balancing the dataset, allowing to reduce presence of label noise in the training set. Another interesting outcome of this experiment has been the comparison of conventional classification in presence of label noise. The comparison highlighted how some methods such as DTs or SVM (which have been considered as viable classification methods for smartphone-based HAR [1]) may not represent an optimal solution when in the presence of label noise.

A multiple-user dataset is, however, required to measure the advantage of personalization against the one-classifier for an all users approach. Similarly, a larger dataset would allow for comparing users with different ways of carrying the smartphone, while, in this experiment, it has been restricted to the most common (trouser pocket).

The next steps will include the collection of a multiple-user dataset to further investigate personalized approaches to HAR. In conclusion, this study provided two main outputs. With respect to the preliminary investigation in [14], it highlights how information on the quality of produced labeling can be beneficial. Finally, the experiment has identified some viable classification methods as being more robust to a dataset subject to label noise.

## Figures and Tables

**Table 1:** Comparison of recent studies on HAR adopting supervised or semi-supervised approach. In most cases dataset is assumed to be fully labeled, while only some studies explored ways to reduce the burden of data annotation. Target Activities (Ly = Lying, Si = Sitting, St = Standing, Wa = Walking, WU = Walking Upstairs, WD = Walking Downstairs, Ru = Running, Be = Bend, Fa = Fall, Da = Dancing, Cy = Cycling, Tr = Transportation, StS = Sit-to-Stand, StL = Sit-to-Lie, LtS = Lie-to-Stand, Tu = Turning, ShT = Sharp Turning).

Ref	Sensors	Target activities	Labeling	Accuracy
Stikic et al. 2011 [12]	accelerometer	Si, St, Wa, Tr, some ADLs	Partial (label propagation)	76%
Siirtola et al. 2012 [15]	accelerometer	Idle (Si/St), Wa, Ru, Cy, Tr	Full	95%
Anguita et al. 2013 [31]	accelerometer	Si, St, Wa, WU, WD	Full	89%
Pei et al. 2013 [32]	accelerometer, gyroscope	Si, St, Wa, Wa (fast), Tu, ShT	Full	92%
Bayat et al. 2014 [33]	accelerometer	Wa (fast), Wa (slow), Ru WD, WU, Da	Full	91%
Cleland et al. 2014 [10]	accelerometer	St, Wa, Ru, Tr (bus)	Partial (user prompt)	85%
Bhattacharya et al. 2014 [34]	accelerometer, gyroscope	Idle (Si/St), Wa, Tr (bus), Tr (tram), Tr (train)	Partial	79%
Reyes-Ortiz et al. 2016 [35]	accelerometer, gyroscope	Ly, Si, St, Wa, WU, WD, StS, StL, LtS	Full	96%
Ronao et al. 2016 [24]	accelerometer, gyroscope	Ly, Si, St, Wa, WU, WD	Full	94%
Hong et al. 2016 [36]	accelerometer	Ly, Si, St, Wa, Cy, Be, Fa	Partial (label propagation)	83%
Hassan et al. 2018 [26]	accelerometer, gyroscope	Ly, Si, St, Wa, WU, WD, StS, StL, LtS	Full	89%
Cao et al. 2018 [37]	accelerometer, gyrsocope	Si, St, Wa, WU, WD	Full	94%
San-Segundo et al. 2018 [38]	accelerometer	Si, St, Wa, WU, WD, Cy	Full	91%

**Table 2:** The set of features used in the experiment.

Domain	Signal	Features
Time	3D Magnitude of Acceleration	Mean, Variance, Min, Max, Range, Skewness, Kurtosis
Time	*x*-, *y*- and *z*-axes of Acceleration	Absolute value of Mean, Variance, Range
Frequency	3D Magnitude of Acceleration	Number of peaks in PSD, Location of highest peak

**Table 3:** Structure of the dataset. For each data point, the first column contains the manually annotated labels while the second column contains the label generated automatically.

Label	Weak Label	Feature 1	⋯	Feature n
TRANSPORTATION	SITTING	$f_{1}^{1}$	⋯	$f_{n}^{1}$
TRANSPORTATION	TRANSPORTATION	$f_{1}^{2}$	⋯	$f_{n}^{2}$
⋯	⋯	⋯	⋯
WALKING	WALKING	$f_{1}^{m}$	⋯	$f_{n}^{m}$

**Table 4:** Number of samples with manual labeling composing the ground truth.

Class	Samples	Hours:Minutes:Seconds
Sitting	395004	56:01:51
Walking	169908	26:09:26
Running	8250	01:20:09
Cycling	19132	02:40:35
Transportation	75244	11:47:21
**Total**	**667538**	**97:59:22**

**Table 5:** Mean values for precision, recall and f-score obtained using 10-fold cross validation for all classifiers.

Algorithm	Precision	Recall	F-Score
Nearest Centroid	0.6816	0.6334	0.6418
DT	0.8249	0.7878	0.7979
Random Forests	0.8666	0.8299	0.8394
kNN	0.8355	0.8079	0.81405
Multi-class SVM	0.7630	0.7414	0.7424
NN 18 × 12 × 6	0.8394	0.8127	0.8188
NN 18 × 36 × 12 × 6	0.8585	0.8345	0.8410

**Table 6:** Confusion Matrix obtained using the NN that measured the highest f-score.

	Sitting	Walking	Running	Cycling	Transportation
**Sitting**	**0.88462**	0.03365	0.00481	0.0625	0.01442
**Walking**	0.03967	**0.79332**	0.04802	0.09395	0.02505
**Running**	0.00785	0.02356	**0.93979**	0.00524	0.02356
**Cycling**	0.01734	0.07514	0.01156	**0.85549**	0.04046
**Transportation**	0.02588	0.03512	0.01109	0.14787	**0.78004**

## Abbreviations

The following abbreviations are used in this manuscript:

ADL	Activity of Daily Living
AR	Activity Recognition
ARC	Activity Recognition Chain
CRF	Conditional Random Field
CNN	Convolutional Neural Network
DBF	Deep Belief Network
DFT	Discrete Fourier Transform
DL	Deep Learning
DT	Decision Tree
EP	Emerging Patterns
FFT	Fast Fourier Transform
HAR	Human Activity Recognition
HMM	Hidden Markov Model
kNN	k-Nearest Neighbors
MIL	Multi-Instance Learning
ML	Machine Learning
NN	Neural Networks
PMML	Predictive Model Markup Language
PSD	Power Spectral Density
SCCRF	Skip-Chain CRF
SVM	Support Vector Machine
